# Encounters and Disagreements Between Indigenous Family Education and School Education: Narrative Reviews

**DOI:** 10.3390/bs15111502

**Published:** 2025-11-05

**Authors:** Fabiola Godoy-Leal, Katerin Arias Ortega, Enrique Riquelme Mella

**Affiliations:** 1 Department of Health, Facultad de Salud, Mendoza, Universidad Santo Tomás, Los Angeles 4430000, Chile; 2 Department of Education, Facultad de Educación, Campus Juan Pablo II, Universidad Católica de Temuco, Temuco 4780000, Chile; eriquelme@uct.cl; 3 Department of Psychology, Facultad Ciencias de la Salud, Campus San Francisco, Universidad Católica de Temuco, Temuco 4780000, Chile; karias@uct.cl

**Keywords:** education, family education, school system, intercultural education

## Abstract

In Chile, school education was established in indigenous territories with the aim of homogenizing knowledge in the education of children within a Western framework; this process has resulted in the denial of the education of indigenous families. The objective of this article is to reflect on the objectives of indigenous family education and school education at all levels for the training of new generations, revealing the potentialities of articulation between both forms of knowledge in education from an epistemological pluralism through a narrative review. To this end, a theoretical and empirical review was carried out in the Scopus, Scielo, and Scholar databases of the last decade. The article’s reflection includes encounters in the family–school relationship in indigenous territory, the involvement in horizontal educational processes for the construction of knowledge, and access to improve the socioeconomic situation of the students. However, there are disagreements with Western schooling that annul the social and cultural aspects of indigenous students’ origin. It concludes by proposing educational strategies such as (a) incorporating indigenous scholars into the teaching–learning processes, (b) contextualizing education, and (c) the articulation of contents and methods specific to the territory in the school system, so that it responds to the ways of teaching and learning from the sociocultural logic itself.

## 1. Introduction

The Chilean school education system originated during the period of colonialism, a period in which several countries in the Americas were governed by European or Western monarchies who, among others, pursued territorial expansion and obtained wealth from indigenous territories (Aizpuru, 2000; Gastélum, 2006; Prévil & Arias-Ortega, 2021). By establishing the republic in the colonized American territories, the logic of instruction of the social, cultural, and spiritual order established by the dominant society, which was materialized through the church and school education, was maintained (Arias-Ortega, 2022; Arias-Ortega et al., 2023). The foundation of missionary schools in American indigenous territories was directed by religious groups to eliminate the ‘indigenous’ of the subjects through Christianity and the annulment of the vernacular language, which promoted the dominant colonizing language (Aizpuru, 2000; Arredondo, 2013; Dillon et al., 2022). In Chile, the school played a fundamental role in the positioning of the State and the unification of the nation, which denies the presence of the indigenous in society (Arias-Ortega et al., 2023). Thus, through school education, it was sought to eliminate the being, knowledge, and actions of the indigenous people.

The implementation of the school education system in the colonized indigenous territories led to the implementation of a schooling process that does not recognize or validate the social and cultural frameworks of indigenous family education in the teaching and learning processes, considering it to have a lower social value, given its primitive and barbaric origin (Battiste, 2005, 2018; Campeau, 2021). This dominant conception of both the indigenous and their epistemic frameworks translates into a series of formative dilemmas in the indigenous student when they encounter a Western Eurocentric teaching that differs from the knowledge of their culture (Arias-Ortega & Quintriqueo, 2020; Arias-Ortega et al., 2023; Battiste, 2018; Black & Hachkowski, 2018; Quilaqueo & Quintriqueo, 2017; Toulouse, 2016; Wetzel, 2023) and the need to respond based on school and family educational expectations.

Currently, according to the 2017 CENSO, 34.2% of the total Chilean population is recognized as belonging to indigenous peoples. This population is distributed throughout the country in the 15 existing regions, with the Metropolitan region and La Araucanía being the places with the highest concentration of indigenous people of the Mapuche ethnic group (Instituto Nacional de Estadisticas de Chile, 2017). Furthermore, according to the latest Enrollment Report in Higher Education in Chile for 2024 (Ministerio de Educación Chile, 2024), 150,953 indigenous students were enrolled in Chilean Universities, being 10.9% of the total students enrolled in higher education. Given this, the Chilean Government postulates that school education is an opportunity to improve the quality of life of young people belonging to indigenous peoples (Ministerio de Educación Chile, 2022).

Between the 1990s and 2000, the Chilean government encouraged the quota system, so State Universities offered limited places for indigenous students to enroll in their undergraduate courses. At the same time, the Indigenous Scholarship was enacted, which provides a stipend for student expenses and student homes for indigenous students while they pursue their higher education (Junta Nacional de Auxilio Escolar y Becas, 2020). Likewise, it has focused on intercultural educational development in the Intercultural Bilingual Education (EIB) project, in which educational establishments with more than 20% of indigenous students must have this educational strategy: to promote vernacular languages such as Aymara, Mapuzungün, Quechua, and Rapa Nui (Educación Intercultural Bilingüe en Chile, 2019).

Thus, Chilean school education has carried out sociopolitical actions to include the indigenous people in the educational system at all levels, from primary education to higher education, offering training that allows for cultural and mental transformation instruction (Rocher, 2011). The school was established as a free education institution to avoid illiteracy in the population, and this allows social and cultural inclusion in the dominant society (Tomasevski, 2003). This is how education is conceived as the only way to climb the social ladder, which is accompanied by the acquisition of a better socioeconomic level for the student’s future. In this way, the student body will have the opportunity, through education, to obtain social mobility and sociocultural inclusion, which gives them a sense of belonging and contribution to the prevailing society (Rocher, 2011), as well as giving them tools to understand and interact with the social and cultural environment where they live. However, all this is established under the cancellation of indigenous family knowledge in the promotion of social and cultural hegemony.

Unfortunately, the situation described above has resulted in the following consequences: (a) the absence of indigenous family education within school education that does not contemplate, at the general level of public policies, family knowledge of the territory in the guidelines of institutional management, such as the Institutional Educational Project, Seal and Mission of the school, Educational Improvement Plan, and School Coexistence Management Plan; and (b) initial teacher training is offered that is out of context with the local, regional, and territorial reality in which new teachers are trained, based on a standardized and homogenizing curriculum in a Western culture. In this way, the possibility of training children and young people, indigenous and non-indigenous, from an intercultural perspective, which gives them the tools and competencies to rethink training from an intercultural educational approach, is denied. Ultimately, the existence of a Western Eurocentric monocultural curriculum, which prevents an epistemological pluralism present in school education, causes indigenous knowledge to be made invisible and denied in the teaching and learning processes (Arias-Ortega & Quintriqueo, 2020; Black & Hachkowski, 2018; Quilaqueo & Quintriqueo, 2017).

The problems mentioned contradict the reality of school education systems, which are characterized by the presence of a great social and cultural diversity of the subjects who are educated there, such as the cultural, linguistic, and socio-religious differences of the indigenous population, peasants, and migrants, among others. For example, in indigenous territories that have been colonized, there is a large number of indigenous students in school education, which requires the need to incorporate their own knowledge into the teaching and learning process. Indigenous knowledge is inductive; it is expressed from the global level to the individual level and is learned from the environment that surrounds them through observation and experimentation, establishing a connection with the environment (Pewawardy, 2002; Serrano et al., 2013). In this way, indigenous knowledge is an individual and social construction based on their own episteme.

The American indigenous episteme includes a fluid and experiential form of knowledge derived from the teachings of indigenous family education and transmitted from generation to generation, characterized by collectivity, reciprocity, and respect (Kenea-Boru, 2020). In this way, indigenous family education occurs in the interaction of children and young people with parents, grandparents, and relatives, who play a fundamental role in maintaining sociocultural identity and in perpetuating their own epistemic practices as a way of keeping their own cosmogonic framework alive (Aguilar, 2001). In this way, through indigenous family education, social memory is transmitted, which is the investigation of the social relations of a community to explain the behaviors related to its knowledge (Quilaqueo, 2012).

At present, school education maintains a power relationship of school knowledge of a Western Eurocentric nature that generates an epistemological distance from indigenous knowledge, causing disagreements to emerge between school education and indigenous family education (Quilaqueo, 2012). The utilitarian logic of Western Eurocentric education constitutes a meeting point between the objectives of school education and the expectations of indigenous family education in relation to the education provided in school (Silva-Peña et al., 2013). These encounters and disagreements present in school education and indigenous family education develop a dynamic and changing educational reality within a Western framework in the educational system that develops between the various actors who interact within territories of social and cultural diversity. Within this framework, it is worth asking the following: What are the encounters and disagreements present in school education and indigenous family education in different colonized societies? What changes are needed in the Chilean school system to promote education with epistemological pluralism?

## 2. Methods

This article discusses different theoretical perspectives regarding family, indigenous, and school education at all levels. A methodology of review of national and international scientific articles on school education and indigenous family education was used, developed through research with an interpretative approach, with data provided within an investigative narrative perspective from a review of empirical research articles (Sukhera, 2022). A narrative review is carried out to describe indigenous experiences of the articles investigated, which allows interpretation and criticism (Sukhera, 2022). This is unlike the systematic review, which is a method that can be biased in the description of indigenous context articles. The selected articles are located within a colonial and postcolonial educational framework of indigenous family education and school education from an indigenous relational paradigm.

The search plan for the selection of articles considered the following procedure: (1) a review of scientific articles was carried out in online databases (Scopus, Scielo and Google Scholar) of national and international journals, expanding the search between 2010 and 2024, in order to have access to bibliography with cultural diversity and an in-depth study of the subject; (2) a review of texts from previous years to those indicated was included, with significant contribution to the article that establish ideologies that have served as a basis for proposing concepts and definitions of the proposed topic; (3) the search was focused on the keywords (a) indigenous family education, (b) school education, and (c) school education in an indigenous context; and (4) those articles that contribute to understanding the object of study on the encounters and disagreements of indigenous family education and school education were selected. Subsequently, articles and books outside the search years that were relevant to the analysis process of the topic investigated were included. The procedure for analyzing texts involves a content analysis to inductively identify the units of meaning about the object of study within a critical paradigm. This is based on the theoretical approach of multi-referentiality proposed by Jaques Ardoíno (2005), who argues for the urgency of epistemological pluralism for the encounter of indigenous family and school education in the teaching and learning processes.

## 3. Results

The objective of this article is to discuss the encounters and disagreements between indigenous family education and school education at all levels promoted by the government to reveal the possibilities of articulation of these two epistemes in the teaching and learning processes, which will allow us to rethink school education from an intercultural epistemological pluralism. The latter is from a perspective of critical interculturality in which elements of indigenous knowledge are incorporated within the Western frameworks of the dominant society (Walsh, 2010). This is based on an indigenous epistemology of intersubjectivity, in which knowledge is relational to the social and cultural context. Nineteen documents with research from different parts of the world were selected (Table 1).

When reading the selected articles, we proceeded to identify those articles that explore the experiences of indigenous students and families regarding institutional school education. During the review of the selected articles, there were themes that were relevant, and the necessary content emerged to answer our research questions. The three emerging themes for our analysis were as follows: (1) school education in an indigenous context, which addresses the definition of school, its objective, and the knowledge used for student learning; (2) indigenous family education, where the contents, methods, and educational purposes for the training of children and young people from their own epistemic frameworks are deepened, and (3) educational encounters and disagreements between indigenous and school knowledge to reinforce the teaching and learning processes, strengthening the sociocultural identity of indigenous and non-indigenous students, which allows generating a multi-referential view of school education in an educational context with social and cultural diversity.

## 4. School Education in an Indigenous Context

School education was established as a State strategy to colonize territories that were occupied by indigenous peoples. These indigenous communities had both a different oral language and customs specific to their culture, which was an impediment to geographical expansion (Pewawardy, 2002). From this perspective, school education emerges as a way of facilitating sociocultural domination, based on a logic of monocultural and Eurocentric thinking typical of the colonizers, to homogenize Western knowledge over indigenous knowledge (Tubino, 2011). School education focuses its teaching on symbolic representations and patterns of Western behavior in order to acquire norms of values, behaviors, and thinking from the dominant society, which has caused ethnic segregation (Arias-Ortega et al., 2018). This is evident in the design of the educational system that is implemented from an economic, social, and cultural disparity between indigenous and non-indigenous students (Arias-Ortega & Quintriqueo, 2020), causing a relationship of asymmetry among students and generating diverse positions among educational actors.

School education in an indigenous context has made it possible to create different meanings for indigenous peoples. We will present some examples that reveal problems present in this topic. In the Borena culture, the schooling of children is a social and economic loss for their family and community because during schooling, they must stop herding their animals, which implies poverty and hunger for their family group (Kenea-Boru, 2020). In a Native Hawaiian community, schooling has caused students of indigenous origin to disconnect from their culture and territory, which makes it difficult to return to their community due to Western teaching that modifies their motivations and their personal conception of identity (Kaomea, 2012). In the Mapuche culture, school education ignores the knowledge of this indigenous group, which causes a loss of sociocultural identity in educational instruction, generating an inequality of opportunities in access to knowledge for children and young people (Arias-Ortega et al., 2023). From this perspective, it is relevant to highlight a multi-referentiality of school education that presents inequalities and economic and geopolitical fractures that affect the educational success of indigenous and non-indigenous schoolchildren, increasing the asymmetries between these two cultural groups (Ardoíno, 2005).

In the educational framework, there are experiences in various countries to try to modify school education and thus adapt it to the indigenous context and improve access to education and knowledge for children and young people of indigenous peoples. In Australia, an example of State school education is one that promotes the interaction of university students with scholars from indigenous contexts so that they integrate local knowledge into their professional training (Bullen et al., 2017). González-Terreros and Torres-Carrillo (2020) point out that there is a need to bring together older and younger people to strengthen the sociocultural belonging of the community. In New Zealand, indigenous educational methods have been incorporated into the university teaching of the Maori culture, taonga tuku iho, which teaches through observation of the environment and promotes reflective learning (Macfarlane et al., 2017). In Mexico, Ecuador, and Bolivia, intercultural universities have been founded, in which the educative curriculum is modified to integrate knowledge of the indigenous culture, where older indigenous people are integrated to promote their own knowledge (Delgadillo, 2018; Krainer et al., 2017; Rosado-May, 2017). In our country, Chile, the Ministry of Education implemented bilingual intercultural education in response to indigenous demands for an education with social, cultural, linguistic, and territorial relevance (Arias-Ortega et al., 2023; Krainer et al., 2017). However, it has not been possible to position a school education in accordance with the indigenous cultural perspective that situates their knowledge within a teaching of knowledge and in a place conducive to it in symmetry with the Western episteme (Arias-Ortega & Quintriqueo, 2020).

In this perspective, Toulouse (2016) and Campeau (2021) argue that the classroom is a space where an exchange of knowledge takes place, it can happen inside and outside a building, and it has students and teachers who participate in a variety of learning relationships. On the contrary, in an indigenous context, families and communities expect formal education to have values that are specific to the society and culture from which the students come in order to allow these young people to adapt to the educational system and prevent them from dropping out of school (Arias-Ortega & Quintriqueo, 2021). In this way, the space must be welcoming, fostering educational coherence and expectations regarding respectful behavior, acceptance of difference, and taking risks in learning to allow a relationship between students and communities that achieve mutual development (Wallace, 2011; Wetzel, 2023). This environment must generate pluralistic learning by integrating knowledge of the student’s family and community context. In addition, it must express the emotional, intellectual, and spiritual domains of holistic education, which must be respected and realized in order to connect students with their culture. Children and young people need a safe space that affirms their diversity and identity, where they can be honored with cultural, linguistic, and affirmative models that support the construction of their knowledge (Toulouse, 2016).

However, in the educational system, the teaching and learning that takes place in the classroom has focused on conceptual learning through the segmentation of educational topics, perpetuating disciplinary knowledge (Monereo et al., 2001). It is in the classroom where indigenous children tend to express their differences with the rest of the student body. Foley (2004) points out the existence of the silent indigenous, relating the experience of schoolchildren from the Mesquakis native community of the United States in Western schooling. The teachers observe that they are shy students; however, they are children who have attitudes of rejection towards a Western education. This study reports experiences in which students sit at the back of the classroom and try not to attract attention because they are in a place dominated by white people. Indigenous students report that they take this attitude out of low self-esteem, boredom, or indifference, and many other times, it is a form of protection from a context that they do not understand. Silence is used by indigenous students to avoid complicated situations in the classroom; for example, avoiding a question asked by the teacher. Some teachers reveal that if they ask an indigenous child and they do not answer, they do not require them to do so. Unlike white students, who are forced to respond, this action generates resentment among students. In view of this, it is essential to avoid the existing prejudices of indigenous students on the part of the teacher.

In this way, school education focuses on the teacher’s methodology to ensure that the contents, topics, and information are learned by the student and the development of competencies is generated (Pamplona et al., 2019). Thus, students are confronted with an education proposed from the top-down verticality, which imposes a point of view of the educator, does not interact with the students, and is authoritarian (Crocker et al., 2004). The teacher exercises a position of domination of power, especially with indigenous students (Foucault, 1969/2002), and at the same time, it generates some privileges and affable actions in front of the white student body that they do not have with the rest (Foley, 2004). From this point, it is necessary to propose actions that favor education with social and cultural relevance.

Given this, teachers and students should generate a relationship in their environment by establishing lines of communication to generate learning that is open and confidential, facilitating the incorporation of the student’s own epistemes (Black & Hachkowski, 2018). Arias-Ortega et al. (2023) and Hachkowski (2011) argue that it is essential for educators to engage in a self-reflection of the transition of their teaching philosophies to incorporate indigenous understanding and knowledge into their curricula and pedagogy. The educator must be able to generate pedagogical transformations between Western and indigenous cultures in order to foster an education with epistemological pluralism, which promotes the inclusion of sociocultural knowledge relevant to teaching (Aikenhead, 2001). In this way, teachers are expected to teach indigenous students an education that incorporates the knowledge of their culture and school knowledge within the same space. Through this education, “it is expected that the new generations will have the possibility of learning the knowledge of the family, and that which is delivered in the process of schooling” (Quilaqueo & Quintriqueo, 2017). This is what Ardoíno (2005) proposes as a partner education, that the teacher is involved in a symmetrical educational relationship with the indigenous student. It is also relevant to include the meaning of school education for indigenous families.

## 5. Indigenous Family Education

Indigenous family education is based on teaching that allows the transmission of knowledge and knowledge of indigenous culture to new generations. Indigenous family education plays a fundamental role in the community and is an essential part of its worldview; it is in this space that children and young people are taught about culture, history, and being indigenous in order to train them in relation to their own epistemological frameworks in the indigenous conception (Arancibia et al., 2014). According to Kaomea (2012) and Christie (2016), indigenous families have roots consisting of an extensive and clanic group, as opposed to what is known as the nuclear family in Western society. This family group maintains its knowledge in a close relationship of the person with the nature and spirituality of his environment (Quilaqueo et al., 2016a).

From this perspective, in indigenous family education, teaching is proposed through its own educational strategies, which refer to the way children and young people are taught from educational actions that underlie their own social and cultural frameworks based on a rationality of the culture of origin (Quintriqueo & Quilaqueo, 2019). Among the educational strategies are (a) stories, which transmit knowledge of local history and values through testimonies by a family member or sociocultural scholar; (b) the family circle is a bodily position of social equality that takes place in a certain space whose purpose is symmetrical communication between the members of a group; and (c) family and social participation, in which children and young people, through the observation and imitation of the members of the family and community, learn sociocultural actions present in the territory and in the sociocultural and socio-religious practices that allow them to develop an attitude and behavior according to their own epistemic framework (Black & Hachkowski, 2018; Quintriqueo & Quilaqueo, 2019).

The story is one of the educational strategies of indigenous societies, which transmits their own knowledge and educational knowledge. It is constructed through words created in a socially agreed-upon language to understand each other within a group. These words are necessary to express that which is infra-superficial that allows knowledge to be transmitted (Ardoíno, 2005; Battiste, 2018; Christie, 2016; Quilaqueo & Quintriqueo, 2017). It is important that family and community stories are passed down from generation to generation (Black & Hachkowski, 2018). It is part of their way of being and acting; through knowing how to listen, they adopt a natural teaching–learning process (Quintriqueo & McGinity Travers, 2009). This account is a vital part of a community to perpetuate its cultural identity.

Another strategy used in indigenous cultures to teach and learn are traditional circles, which allow the individual, through their spatio-temporal location, to assume an attitude of respect, status, and time to share their opinions with all members of the group, in which circularity favors face-to-face interaction (Black & Hachkowski, 2018; Kenea-Boru, 2020). In this way, a position of equality is achieved among all members of the community, facilitating the exchange of knowledge and contributing to a co-construction of knowledge. Indeed, developing teaching and learning processes from the logic of family education implies that the members of the community are educational agents, so that the knowledge to be transmitted acquires a notion of a psychosocial and sociological nature that requires another to exist (Ardoíno, 2005). This is how the traditional circle represents an education with horizontality, in which knowledge is built symmetrically. This differs from vertical education, in which the teacher takes a role of power of knowledge over other members, which is proposed by the Western educational system (Crocker et al., 2004).

The next educational strategy that we present is the family and social participation of indigenous communities, in which community learning is promoted to transmit knowledge to new generations. Ardoíno (2005) defines learning as a pedagogy that is applied to the functionality of the child through rational practices that teach know-how but that excludes a know-how. Indigenous families promote know-how through social and cultural participation, as in the Borena in Ethiopia, where indigenous family education is based on instilling good customs, respect for their environment, and promoting their main economic and social activity, pastoralism (Kenea-Boru, 2020). The educational purpose of indigenous family participation is to teach through experience the main sociocultural activities of the community. This education is similar to that presented in the Wichi culture in Argentina, where community life is essential for them, as well as respecting and participating in their traditions and collaborating in the activities of gathering, hunting, and cultivating the land (Ossola, 2010). Likewise, in the Mapuche culture in Chile, indigenous family education aims to form good people who are respectful and characterized by having a close relationship with the environment that surrounds them (Alarcón et al., 2018; Quilaqueo et al., 2016a).

Children belonging to an indigenous community learn from the patterns of the community, the relationship with the territory, ancestral knowledge and myths, community values, and the knowledge accumulated in social memory (Molina-Bedoya & Tabares-Fernández, 2014). Children and young people have as an educational strategy the careful observation of actions and events; they repeat processes that allow them to later reflect and examine all the implications and solutions to the problems presented (Black & Hachkowski, 2018; Serrano et al., 2013). In this way, they build their knowledge and their identity, which allows them to understand life from social memory in order to project their personal aspirations and efforts (Quilaqueo & Quintriqueo, 2017). In this perspective, the environment is a facilitating medium for the process of building knowledge of indigenous children and young people, which differs from the conception of the teaching and learning processes that have been installed in school education in an indigenous context. In view of this, it is essential to address the aspirations that the indigenous community has regarding the role of school education.

## 6. Aspirations of School Education from the Indigenous Conception

The aspirations of school education from the indigenous conception have manifested themselves in various ways over time. Initially, Western school education has sought to replace the transmission of knowledge from indigenous families, which has been an inconvenience to maintaining their traditions (Kaomea, 2012). In view of this, it is necessary to explore the meanings that indigenous families have about schooling.

Within the encounters of Western school education that have been imposed in indigenous territories, there are interactions between these actors who have experienced schooling as a positive aspect for their personal development. Kaomea (2012) and Kenea-Boru (2020) point to a tendency to value education as access to a modern world. In this way, parents state that they do not want their children to live experiences of poverty that they have lived. At the same time, there are parents who see schooling as part of personal success but differ in their visions; for some, success is obtaining a professional degree, and for others, it is giving back to the community and their family as essential care within their sociocultural framework (Kaomea, 2012; Silva-Peña et al., 2013).

Another positive aspect of school education is social connotation, in which the indigenous family that is inserted in a Western world relies on the school to take care of its children while they work (Kaomea, 2012). In this educational space, children and young people socialize at school, have fun with others, and, in turn, receive some educational materials that allow them to develop properly as students and access a professional degree (Ossola, 2010; Silva-Peña et al., 2013). In addition, the existence of school education close to the territory allows them to have access to Western Eurocentric knowledge without having to leave their home to learn (Ossola, 2010).

As for the disagreements, there is a loss of family and cultural identity of the indigenous student when they enter school education due to the hegemony of Western Eurocentric epistemology in the teaching and learning processes. According to Ardoíno (2005), epistemology is the philosophy of knowledge that designates the conditions and ways of establishing the validity of this knowledge. The existence of a dichotomy between the epistemology present in indigenous cultures in relation to the epistemology proposed by school education limits a personal construction of the indigenous student, who must adapt to a context and annul his or her own identity (Kenea-Boru, 2020). Indigenous students feel the pressure to separate themselves from their sociocultural heritage in order to try to please and integrate into other customs to avoid being denigrated and labeled by other students and teachers (Beltrán-Cuevas et al., 2019; Christie, 2016). These indigenous young people experience epistemological tensions when they have to develop a double educational rationality; on the one hand, they have a family education in which their own knowledge and educational knowledge are transmitted to them, and on the other hand, they are students in an educational space in which the dominant national culture prevails, which generates a loss of identity, loss of a sense of belonging, and sociocultural dilemmas in students when they have to move between two worlds, in which Western Eurocentric rationality generally prevails.

Other disagreements that exist in schooling are related to the teaching of abstract content that is not useful for students when carrying out actions typical of their community, such as hunting or fishing, and they feel that they waste time in school education (Rosado-May, 2017). In addition, in some situations, school education distances them geographically from their homes, and they have to leave their families to attend boarding schools. This generates economic problems and a disconnection from their culture (Beltrán-Cuevas et al., 2019; Christie, 2016). Indigenous students feel a marked difference when they reach a dominant schooling that causes them to see themselves as an other, generating an otherness in the educational system (Ossola, 2010). This other is understood as different and that it has established limits, but it does not limit an other person in any way, and that can count on a heterogeneous nature, which is not necessarily recognized and validated in school education (Ardoíno, 2005). In a school environment, it can lead to the presence of prejudiced and stereotyped relationships of indigenous students or social minority groups, which can cause emotional isolation that could negatively affect their academic performance (Arias-Ortega & Quintriqueo, 2020).

From this perspective, school dropout is high among indigenous students compared to non-indigenous students as school level increases (Kenea-Boru, 2020). When inquiring about the causes of school dropouts, there are some problems, such as (a) economic—lack of resources to acquire educational materials, food, and maintenance expenses; (b) distance—distance from their homes to attend schools present in urban areas or other territories; (c) language barrier—they must cancel their mother tongue in order to acquire the prevailing language of the culture where the educational establishment is located; and (d) self-exclusion on the part of the student due to inaccessible teaching or form of knowledge from others (Paladino, 2019). And in more complex cases, indigenous youth are forced to drop out of school education due to the absence of their father, due to imprisonment for indigenous demands, making access to the education system even more difficult (Comisión Interamericana de los Derechos Humanos, 2012).

In conclusion, indigenous families have encountered schooling as an institution of social and economic support, which also has a sociocultural value for achieving academic success and improving their quality of life. Among the disagreements, there is the cultural annulment of students and the forced adaptation to another culture different from the one of origin, thus affecting their sociocultural identity and negatively affecting the continuity of indigenous family education, which results in the loss of their own knowledge and educational knowledge.

## 7. Discussion

According to the literature reviewed, it is observed that there is a dichotomy between indigenous family education and school education that produces encounters and disagreements in students of indigenous origin who attend the formal education system. According to Álvarez et al. (2011), the family is the natural and primary educational agent and, therefore, is fundamental. The family has the right to choose for their children the education that responds to their philosophical, ethical, and religious convictions. However, society has imposed a monocultural school education, which imposes a single type of thinking; it is also considered factual because the school represents an institution that promotes teaching that prepares for life (Ardoíno, 2005). At the same time, it annuls the knowledge of indigenous students and perpetuates educational homogenization, imposing a single socially accepted point of view (Battiste, 2018).

In the current educational scenario, indigenous students are exposed to a homogenization of Western knowledge and educational strategies that has caused their culture to be seen as backward and has transformed them into passive recipients of Western Eurocentric knowledge (Battiste, 2005, 2018). These educational strategies differ from those learned within their family; however, students must be able to articulate other ways of learning. According to Ardoíno (2005), at this time, “negatricity” emerges, defined as the ability of people to disarm the strategies that are imposed by a dominant society within schooling.

In this sense, it is necessary to consider a school education that is capable of articulating different knowledge in the same context, allowing heterogeneity among students through intercultural epistemological pluralism. Ardoíno (2005) states that heterogeneity should be understood as what another is for one to the extent that he resists it; this other, who is different, should not be a limit for those around him but rather presents a heterogeneous nature. In this way, it would be expected that school education would contemplate a vision with educational epistemological pluralism to allow indigenous students to feel part of the educational system and their knowledge to be socioculturally accepted According to Lara-Guzmán (2015), education must be able to dialogue the points that hinder each other, as well as to bridge the abyss between the knowledge of the institution, the knowledge of indigenous family education, and the actions developed in the classroom. School education should be used to acquire knowledge that generates encounters with the knowledge of indigenous communities present in the territory, taking into account the possibility of educational, social, cultural and territorial problems that could trigger disagreements between both educational actors, which poses the challenge of addressing an intercultural epistemological pluralism in school education in indigenous territories.

Between the encounters of indigenous family education and school education, there is a social dimension that is important both for the family and for the indigenous student related to socioeconomic ascent. Relevance is given to the sense of school and to be part of the dominant society, learning Western knowledge that allows them to integrate into the sociocultural and economic system where they will develop as people (Lara-Guzmán, 2015). However, inserting themselves into Western schooling establishes certain patterns of behavior and thoughts that differ from those learned in their family, causing a loss of identity and generating disagreements with the origin of their indigenous family education (Christie, 2016). In this way, they must adapt to a school system that uses Western strategies and annuls indigenous family educational strategies, in which students must disconnect from their culture and generate a double rationality of thinking to be accepted by the different actors in the education system.

Double rationality is a personal educational strategy of students of indigenous origin or from contexts of social and cultural diversity to adapt, in which students must use two logics, the indigenous and the school, to learn in the school environment (Quilaqueo et al., 2016b). This double rationality is promoted by indigenous parents to resist the sociocultural domination of the State (Arancibia et al., 2014). In this way, a double rationality present in the indigenous student can be part of a Western educational system, comprising a sense of schooling that articulates indigenous family education and school education from an intercultural epistemological pluralism.

## Figures and Tables

**Table 1:** Documents reviewed.

Autor (Year)	Article	Journal	Country
Arias-Ortega and Quintriqueo (2020)	La educación superior en el contexto mapuche: El caso de La Araucanía, Chile.	Revista Electrónica Educare	Chile
Kenea-Boru (2020)	Education for children in the Borena pastoral community in Ethiopia: practices and challenges.	Ethiopian Journal of Education and Science	Etiopía
Kaomea (2012)	Reconceptualizing Indigenous Parental Involvement in Early Educational Settings: Lessons from Native Hawaiian Preschool Families.	International Journal of Indigenous Politics	Hawai, USA
Bullen et al. (2017)	What predicts health students’ self-reported readiness to work in Indigenous health settings?	The Australian Educational Researcher	Australia
González-Terreros and Torres-Carrillo (2020)	Educación popular y educación propia: diálogos desde experiencias educativas en el Cauca	Revista Colombiana de Educación	Colombia
Macfarlane et al. (2017)	Social and emotional learning and Indigenous ideologies in Aotearoa New Zealand: a biaxial blend.	Capítulo de Libro	Nueva Zelanda
Delgadillo (2018)	“Iyambae”: in search of an emancipatory higher education in the UNIBOL Guaraní and Lowland Peoples.	Alteridad	México
Krainer et al. (2017)	Educación superior intercultural y diálogos de saberes: el caso de los Amawtay Wasi en Ecuador.	Revista Educación Superior	Ecuador
Rosado-May (2017)	Formación universitaria intercultural para indígenas mayas de Yucatán.	Antropológica	México
Arias-Ortega and Quintriqueo (2021)	Relación educativa entre docente y educador tradicional en educación intercultural bilingüe.	Revista Electrónica de Investigación Educativa	Chile
Wallace (2011)	Power, practice, and a critical pedagogy for non-Indigenous allies.	Canadian Journal of Native Studies.	Canadá
Hachkowski (2011)	Aboriginal education: a transition of worldviews.	Capítulo de Libro	Canadá
Arancibia et al. (2014)	Análisis de la significación que los estiudiantes universitarios de poblaciones indígenas otorgan a su ingreso a la educación terciaria.	Capítulo de Libro	Chile
Christie (2016)	Fostering the Educational Movement: Connecting University Writing Classrooms on Oahu with Indigenous Family Values and Family Learning for Immigrants.	Pacific Asia Inquiry	Hawai, USA
Quilaqueo et al. (2016a)	Educación mapuche y educación escolar en la Araucanía ¿doble racionalidad educativa?	Cadernos de Pesquisa	Chile
Ossola (2010)	Educación superior de jóvenes indígenas en la provincia de Salta (Argentina): trayectorias personales, tensiones familiares y expectativas comunitarias.	Capítulo de Libro	Argentina
Molina-Bedoya and Tabares-Fernández (2014)	Educación propia. Resistencia al modelo de homogenización de los pueblos indígenas de Colombia	Revista Latinoamericana	Colombia
Beltrán-Cuevas et al. (2019)	Universitarios indígenas en el área de la salud: una aproximación a la realidad.	Revista Mexicana de Medicina Forense y Ciencias de la Salud.	México
Paladino (2019)	Pueblos indígenas y educación superior en Argentina. Datos para el debate.	Revista ISEES (Inclusión Social y Equidad en la Educación Superior)	Argentina

Source: Prepared by the authors.

## Data Availability

This study did not collect any data.
